# Do disease-modifying antirheumatic drugs and non-steroidal anti-inflammatory drugs increase the burden on ankylosing spondylitis patients with mild-moderate COVID-19? evidence from a retrospective cohort study

**DOI:** 10.3389/fphar.2023.1266915

**Published:** 2023-10-30

**Authors:** Yan Li, Zhengyuan Hu, Yufei Guo, Zheng Zhao, Kunpeng Li, Xiuru Wang, Jie Zhang, Dongfeng Liang, Jianglin Zhang, Xiaoyue Hu, Jian Zhu, Feng Huang

**Affiliations:** ^1^ Department of Rheumatology and Immunology, The First Medical Center, Chinese PLA General Hospital, Beijing, China; ^2^ School of Social Development and Public Policy, Fudan University, Shanghai, China

**Keywords:** coronavirus disease 2019 (COVID-19), ankylosing spondylitis, TNF-inhibitor, DMARDs (synthetic), cohort study, NSAID (non-steroidal anti-inflammatory drug)

## Abstract

**Objectives:** The impact of non-steroidal anti-inflammatory drugs (NSAIDs), conventional synthetic disease-modifying antirheumatic drugs (csDMARDs) and tumor necrosis factor inhibitors (TNFi) on the outcomes of mild-moderate COVID-19 in patients with ankylosing spondylitis (AS) remains unclear. This study aimed to evaluate the effects of NSAIDs, csDMARDs, and TNFi on AS patients with mild-moderate COVID-19.

**Methods:** This cohort study utilized patient-reported PCR/antigen tests to determine the occurrence of COVID-19 and assessed clinical manifestations to determine its severity. The study focused on two primary outcomes: an increased number of COVID-19 symptoms and a prolonged disease course (longer than 10 or 28 days). Modified Poisson regression was performed to analyze the association between exposures and outcomes.

**Results:** A total of 521 patients were included in the analysis. The median age was 34.8 (inter-quartile range: 27.2–46.7), with 420 (80.6%) being men. Among the patients, 52 (10.0%) had comorbidities and 443 (85%) had been vaccinated. After adjusting for confounding factors, there was no significant association between csDMARDs or TNFi and the presence of more than 5 symptoms in mild-moderate COVID-19 (adjusted relative risk (RRa) 1.08, 95% CI: 0.84–1.40 or 1.09, 0.92–1.29 for csDMARDs or TNFi, respectively), whereas the prevalence of experiencing more than 5 symptoms increased in patients with NSAID monotherapy (RRa 1.22, 95% CI: 1.01–1.46). Similarly, there was no significant association with having more than 10 symptoms (RRa 0.65, 95% CI: 0.26–1.64; 0.95, 0.36–2.54; and 1.01, 0.53–1.91 for NSAIDs, csDMARDs, and TNFi, respectively). Patients who had pre-existing use of NSAIDs, csDMARDs and TNFi had similar odds of experiencing a disease course longer than 10 days (RRa 1.17, 95% CI: 0.82–1.66; 1.18, 0.78–1.77; and 1.22, 0.92–1.63 for NSAIDs, csDMARDs, and TNFi, respectively) and longer than 28 days (RRa 0.94, 95% CI: 0.31–2.81; 0.97, 0.25–3.74 and 1.05, 0.44–2.49, respectively) compared to those not using medication.

**Conclusion:** AS patients treated with csDMARDs or TNFi did not show inferior outcomes in terms of symptom burden or recovery compared to those not using medication in mild-moderate COVID-19. The observed inverse association between pre-existing NSAIDs use and COVID-19 symptom burden in AS deserves further investigation.

## 1 Introduction

Despite the World Health Organization (WHO) declaring an end to the COVID-19 pandemic as a public health emergency (Graham, 2023), it had a significant impact on individuals with chronic inflammatory diseases such as ankylosing spondylitis (AS). This is particularly true for those taking immunomodulatory or immune-suppressive medications known as conventional synthetic or biological disease-modifying antirheumatic drugs (cs/bDMARDs), in addition to the compromised immune system associated with AS itself (Deodhar et al., 2022). Over the past 4 years, new variants of the virus have emerged, which exhibit increased transmissibility but fortunately, have been found to be less virulent than the original virus (Nyberg et al., 2022). For the majority of patients, COVID-19 presents as a mild or moderate disease, with 70%–80% of those infected experiencing mild flu-like symptoms and not requiring hospitalization, even during the early stages of the pandemic (Kun et al., 2023). Previous research has predominantly focused on severe outcomes of COVID-19 in patients with AS, such as hospitalization, admission to intensive care units, mechanical ventilation, and death. Therefore, the outcomes and predictive factors of severe COVID-19 in AS patients have been well-documented (Gianfrancesco et al., 2020; Strangfeld et al., 2021). However, there is a lack of reporting on the outcomes of mild-moderate COVID-19 in AS patients, even though these cases make up the majority of patients during the pandemic. Additionally, it remains unknown whether AS patients are at a heightened risk of experiencing increased symptoms or prolonged recovery periods with mild-moderate COVID-19.

Tumor necrosis factor inhibitor (TNFi) is a widely used bDMARD in the treatment of AS and is known for its immunosuppressive properties. Traditionally, TNFi has been associated with an increased risk of infection (Wroński and Fiedor, 2019). However, during the pandemic, TNFi has been suggested as a treatment option for individuals with severe COVID-19 due to its anti-inflammatory characteristics (Guo et al., 2022). Recent studies have reported that TNFi is associated with reduced odds of severe COVID-19 in people with axial spondyloarthritis, but its impact on individuals with AS and mild-moderate COVID-19 has not been extensively studied (Gianfrancesco et al., 2020; Machado et al., 2023). The objective of this study is to evaluate the effects of csDMARDs and TNFi on the outcomes of individuals with mild-moderate COVID-19. Improving our understanding of these effects will help fill gaps in knowledge regarding the outcomes of AS patients with COVID-19 andmore importantly, provide evidence for modifying the treatment strategy for AS.

## 2 Materials and methods

### 2.1 Study design and patients

This retrospective cohort study was conducted at the outpatient rheumatology clinics of the First Medical Center of the Chinese People’s Liberation Army (PLA) General Hospital, a tertiary referral center in Beijing, China. Patients attending the clinics were invited to participate in the study and complete questionnaires that included demographic data, AS disease characteristics, and COVID-19 infection details. Besides, evaluation of AS disease activity (Bath Ankylosing Spondylitis Disease Activity Index, BASDAI) (Garrett et al., 1994), function level (Bath Ankylosing Spondylitis Functional Index, BASFI) (Calin, et al., 1994) and physical mobility (Bath Ankylosing Spondylitis Metrology Index, BASMI) (Jenkinson et al., 1994) were conducted by a fellowship-trained physician.

COVID-19 infection details included the results of SARS-CoV-2 PCR or antigen tests, vaccination status, COVID-19 symptoms, and the time taken for patients to recover. Patients aged 18 years or older were enrolled in the study from 20 December, 2022, to 31 March, 2023, if they met the 1984 modified New York criteria for AS (van der Linden et al., 1984) and had mild-moderate COVID-19. Patients were identified as having COVID-19 if they had a positive SARS-CoV-2 PCR or antigen test, and the day of the positive test was considered as the index day. The severity of COVID-19 was determined based on the Chinese Diagnosis and Treatment Protocol for COVID-19 (Trial Version ten) (General Office of the National Health Commission, 2023). Mild COVID-19 was defined as patients having upper respiratory infection symptoms as their predominant manifestation, such as fever, cough, or sore throat. Moderate COVID-19 was defined as patients having persistent fever, cough, or dyspnea, but without any of the following signs: respiratory rate ≥30 times per minute, oxygen saturation ≤93% when breathing ambient air, PaO2/FiO2 ≤ 300 mmHg, or lung infiltrates >50% area on images. Patients who denied having COVID-19 or whose AS medications were something other than non-steroidal anti-inflammatory drugs (NSAIDs), TNFi, and csDMARDs were excluded from the study. We retrospectively reviewed exposure variables before the index day and prospectively explored their influences on outcomes.

This study was conducted in accordance with the Strengthening the Reporting of Observational Studies in Epidemiology (STROBE) guidelines and complied with the Declaration of Helsinki.

### 2.2 Exposure variables

NSAIDs, TNFi, and csDMARDs exposure was defined as patients being prescribed NSAIDs, TNFi, and csDMARDs within 12 months before the index date, respectively. TNFi included etanercept and its biosimilars, adalimumab, and infliximab. The range of csDMARDs in this study included sulfasalazine, methotrexate, leflunomide, thalidomide, and iguratimod. Given the clinical practice and the real-world background of the study, patients taking AS medications were grouped as NSAIDs monotherapy, NSAIDs + csDMARDs, csDMARDs monotherapy, and TNFi (with or without NSAIDs/csDMARDs).

Covariates such as age, sex, comorbidity, body mass index (BMI), smoking, alcohol consumption, and vaccination status might be associated with different outcomes in COVID-19. Therefore, they were considered confounding factors and adjusted for in further multivariable analysis. Comorbidities included diabetes, cardiovascular disease (CVD, including hypertension), and chronic obstructive lung disease (COPD). Vaccination status was classified as unvaccinated, partially vaccinated (one dose of inactive vaccine), fully vaccinated (two doses of inactive vaccine or one dose of adenovirus vaccine), and booster vaccinated (three doses of inactive vaccine or two doses of adenovirus vaccine).

### 2.3 Outcomes

The two key outcomes in this study were symptom burden and disease course. Fifteen symptoms related to COVID-19 were collected, including fever (peak temperature >37.3°C), chill, sore throat, hoarse voice, cough, nasal congestion, runny nose, headache, dizziness, dyspnea, myalgia, otologic symptoms, palpitation, abdominal pain, and diarrhea. Following a previous study (Hopkinson et al., 2021), we considered the number of self-reported COVID-19 symptoms as a proxy for disease burden and did not attempt to weigh different symptoms. In this study, the presence of more than 5 symptoms or 10 symptoms was arbitrarily categorized as increased symptom burden at two levels.

Defining long COVID in AS is challenging due to the lack of a globally accepted definition and the overlap of symptoms between long COVID and AS, such as fatigue and arthralgia (Baimukhamedov, 2023). In this study, we calculated the period between the index day and the day when patients reported returning to their “usual health.” Patients with a period longer than 10 days (LC10) or 28 days (LC28) were defined as having long COVID, to different extents.

### 2.4 Statistical analysis

In the descriptive analysis, we assessed the differences in proportions and medians of variables between the exposure group and the unexposed group using chi-squared tests for categorical variables and *t*-tests or Mann-Whitney *U* tests for continuous variables. Missing data were addressed using multiple imputations with five iterations, assuming that the data were missing at random.

The main analysis compared baseline medication exposure *versus* no medication use on COVID-19 symptom burden and disease course. First, to evaluate the association between COVID-19 symptom burden and baseline medication exposure, we used modified Poisson regression with a robust (sandwich) estimation of variance (which allows for binary variables) (Zou, 2004) to calculate the relative risk (RR) and 95% confidence interval (CI). We performed multivariable analysis to adjust for confounding factors, including age, gender, comorbidity, overweight (BMI >25) (Carnethon et al., 2012), smoking, alcohol consumption, and vaccination status. Similar analyses were conducted for the outcome of long COVID. Subsequently, we subgrouped patients treated with TNFi into TNFi monotherapy or combination with NSAIDs or csDMARDs to further explore the influence of TNFi on COVID-19 outcomes using the same approach. Forest plots were used to visualize the results using the R statistical program (Ver 4.0.3) with the forest plot package. Other statistical analyses were performed using SPSS Statistics (version 22; IBM Corp.). A *p*-value <0.05 was considered statistically significant for all analyses.

## 3 Results

### 3.1 General information

**FIGURE 1:**
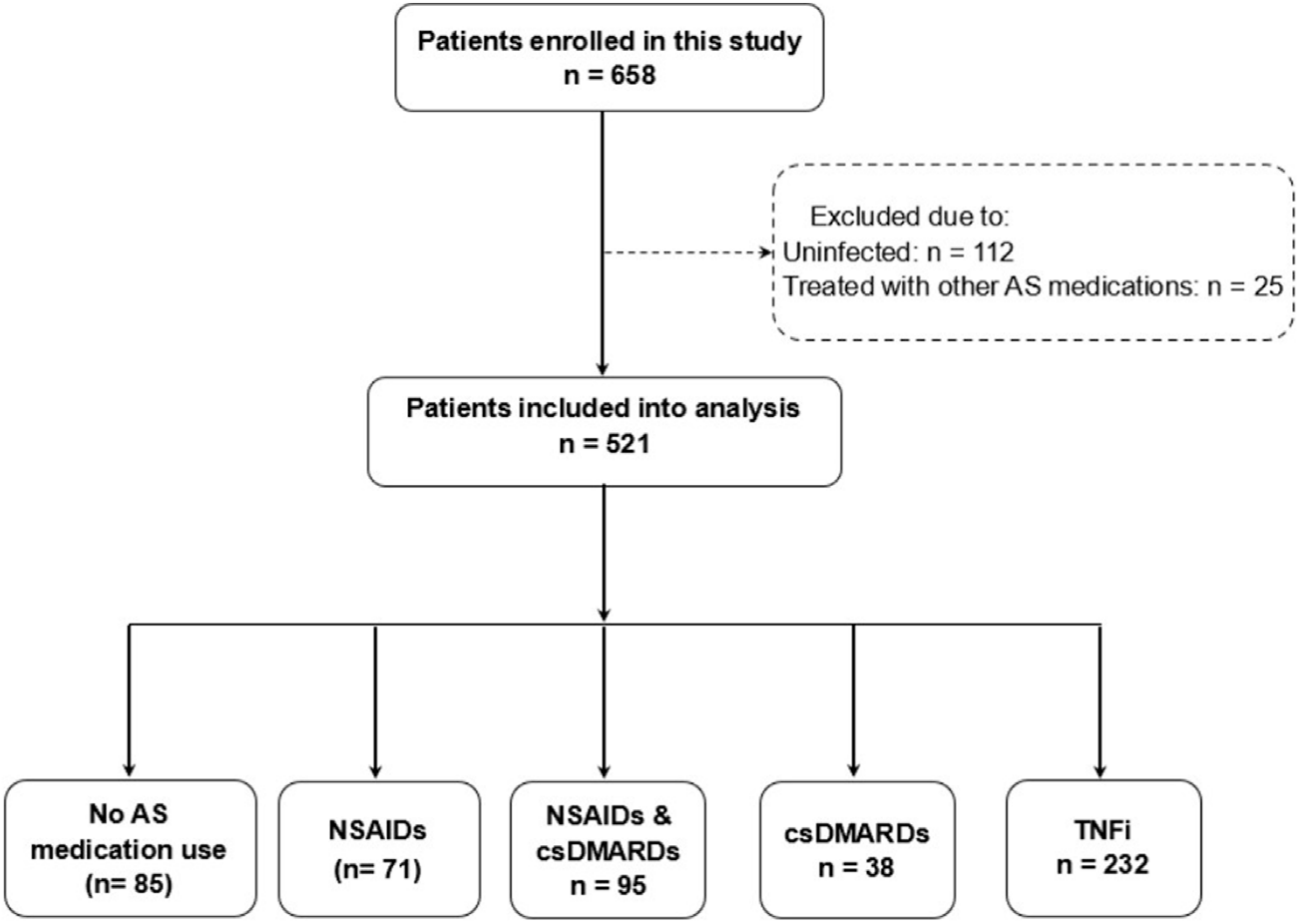
Flow-chart of analytical approach. Abbreviation: NSAIDs, non-steroidal anti-inflammatory drugs. csDMARDs, conventional systhetic disease-modifying antirheumatic drugs. TNFi, Tumor necrosis factor inhibitors.

Questionnaires were collected from 658 AS patients, out of which 112 patients reported negative results for COVID-19 infection. Additionally, 25 patients were taking other AS medications, including 11 patients using Secukinumab, 6 patients using Tofacitinib, and 8 patients using traditional Chinese medicines. These patients were excluded from the analysis, leaving a total of 521 patients (Figure 1). Among these patients, the median age was 34.8 (interquartile range, IQR: 27.2–46.7). Of the 521 patients, 420 (80.6%) were male, 52 (10.0%) had at least one comorbidity, 442 (84.8%) had HLA-B27 positivity, and 443 (85%) had been vaccinated (Table 1).

**TABLE 1:** Baseline characteristics of the participants.

	Total (n = 521)	AS treatment				
		None (n = 85)	NSAIDs (n = 71)	NSAIDs & csDMARDs (n = 95)	csDMARDs (n = 38)	TNFi (n = 232)
Male sex	420 (80.6%)	66 (77.6%)	55 (77.5%)	77 (81.1%)	26 (68.4%)	196 (84.5%)
Age, years						
18–35	290 (55.7%)	48 (56.5%)	39 (54.9%)	55 (57.9%)	19 (50.0%)	129 (55.6%)
36–45	165 (31.7%)	28 (32.9%)	24 (33.8%)	29 (30.5%)	13 (34.2%)	71 (30.6%)
46–55	47 (9.0%)	4 (4.7%)	7 (9.9%)	9 (9.5%)	1 (2.6%)	26 (11.2%)
≥55	19 (3.7%)	5 (5.9%)	1 (1.4%)	2 (2.1%)	5 (13.2%)	6 (2.6%)
BMI	24.5 (22.1, 27.1)	23.9 (21.3, 25.9)	25.3 (22.3, 27.3)	24.6 (22.0, 27.0)	23.8 (21.9, 25.9)	24.6 (22.6, 27.1)
Overweight	233 (44.7%)	31 (36.5%)	38 (53.5%)	42 (44.2%)	14 (36.8%)	108 (46.6%)
Comorbidities						
None	469 (90.0%)	77 (90.6%)	63 (88.7%)	83 (87.4%)	32 (84.2%)	214 (92.2%)
Diabetes	8 (1.5%)	2 (2.4%)	1 (1.4%)	2 (2.1%)	1 (2.6%)	2 (0.9%)
CVD	41 (7.9%)	8 (9.4%)	4 (5.6%)	11 (11.6%)	4 (10.5%)	14 (6.0%)
COPD	10 (1.9%)	1 (1.2%)	4 (5.6%)	1 (1.1%)	1 (2.6%)	3 (1.3%)
Smoking status						
None	328 (63.0%)	51 (60.0%)	46 (64.8%)	69 (72.6%)	24 (63.2%)	138 (59.5%)
Ever smokers	193 (37.0%)	34 (40.0%)	25 (34.2%)	26 (27.4%)	14 (36.8%)	94 (40.5%)
Alcohol consumption						
None	221 (42.4%)	38 (44.7%)	33 (46.5%)	48 (50.5%)	19 (50.0%)	83 (35.8%)
With drinking habit	300 (57.6%)	47 (55.3%)	38 (53.5%)	47 (49.5%)	19 (50.0%)	149 (64.2%)
HLA-B27 (+)	442 (84.8%)	74 (87.1%)	59 (83.1%)	81 (85.3%)	30 (78.9%)	198 (85.3%)
Vaccination status						
Unvaccinated	78 (15.0%)	13 (15.3%)	9 (12.7%)	13 (12.7%)	7 (18.4%)	36 (15.5%)
Partially	19 (3.6%)	0	2 (2.8%)	3 (3.2%)	0	14 (6.0%)
Fully	131 (25.1%)	20 (23.5%)	15 (21.1%)	22 (23.2%)	12 (31.6%)	62 (26.7%)
Booster	293 (56.2%)	52 (61.2%)	45 (63.4%)	57 (60.0%)	19 (50.0%)	120 (51.7%)
BASDAI	2.5 (1.2, 4.0)	3.0 (1.6, 4.4)	3.0 (1.4, 4.2)	2.1 (1.0, 3.5)	2.2 (1.2, 3.8)	2.2 (1.2, 4.0)
BASFI	1.1 (0, 3.2)	1.2 (0.1, 4.4)	1.8 (0.2, 4.8)	0.6 (0, 2.7)	1.4 (0, 2.2)	1.1 (0, 2.9)
BASMI	3.0 (0, 5.0)	4.0 (0, 5.0)	4.0 (0, 5.0)	1.0 (0, 5.0)	3.0 (0, 6.3)	3.0 (0, 5.0)

Data are n (%) for categorical variables and median (IQR) for continuous variables, respectively. Percentages might not sum to 100% due to rounding. Abbreviation: NSAID, non-steroidal anti-inflammatory drugs; csDMARD, conventional synthetic DMARD; TNFi, tumor necrosis factor inhibitor; BMI, body mass index; COPD, chronic obstructive pulmonary disease; cardiovascular disease (CVD, including hypertension).

Regarding AS treatments, 71 patients (13.6%) underwent NSAIDs monotherapy, 95 (18.2%) received NSAIDs in combination with csDMARDs, 38 (7.3%) had csDMARDs monotherapy, 232 (44.5%) were treated with TNFi, and 85 (16.3%) did not receive any of the mentioned medications. Compared to patients who reported no AS medication usage, patients who received TNFi were more likely to be men (196 [84.5%] vs 66 [77.6%]) and less likely to be older than 55 years (6 [2.6%] vs 5 [5.9%]). Patients with NSAIDs monotherapy were more likely to be overweight (38 [53.5%] vs 31 [36.5%]) and have COPD (4 [5.6%] vs 1 [1.2%]), but less likely to be older than 55 years (1 [1.4%] vs 5 [5.9%]). On the other hand, patients with csDMARDs monotherapy were more likely to be older than 55 years (5 [13.2%] vs 5 [5.9%]), but less likely to be male (26 [68.4%] vs 66 [77.6%]). Besides, patients treated with csDMARDs monotherapy, NSAIDs & csDMARDs or TNFi had lower BASDAI (median and IQR was 2.2, (1.2, 3.8), 2.1 (1.0, 3.5) and 2.2 (1.2, 4.0), respectively) and BASMI (3.0 (0, 6.3), 1.0 (0, 5.0) and 3.0 (0, 5.0), respectively) than patients without previous medication use (3.0 (1.6, 4.4) for BASDAI and 4.0 (0, 5.0) for BASMI), whereas patients with NSAIDs monotherapy had worse BASFI (1.8 (0.2, 4.8)) and comparable BASDAI (3.0 (1.4, 4.2)), BASMI (4.0 (0, 5.0)) than patients without previous medication use ((3.0 (1.6, 4.4) for BASDAI, 4.0 (0, 5.0) for BASMI and 1.2 (0.1, 4.4) for BASFI) (Table 1).

### 3.2 Association of AS medications with COVID-19 disease burden

**FIGURE 2:**
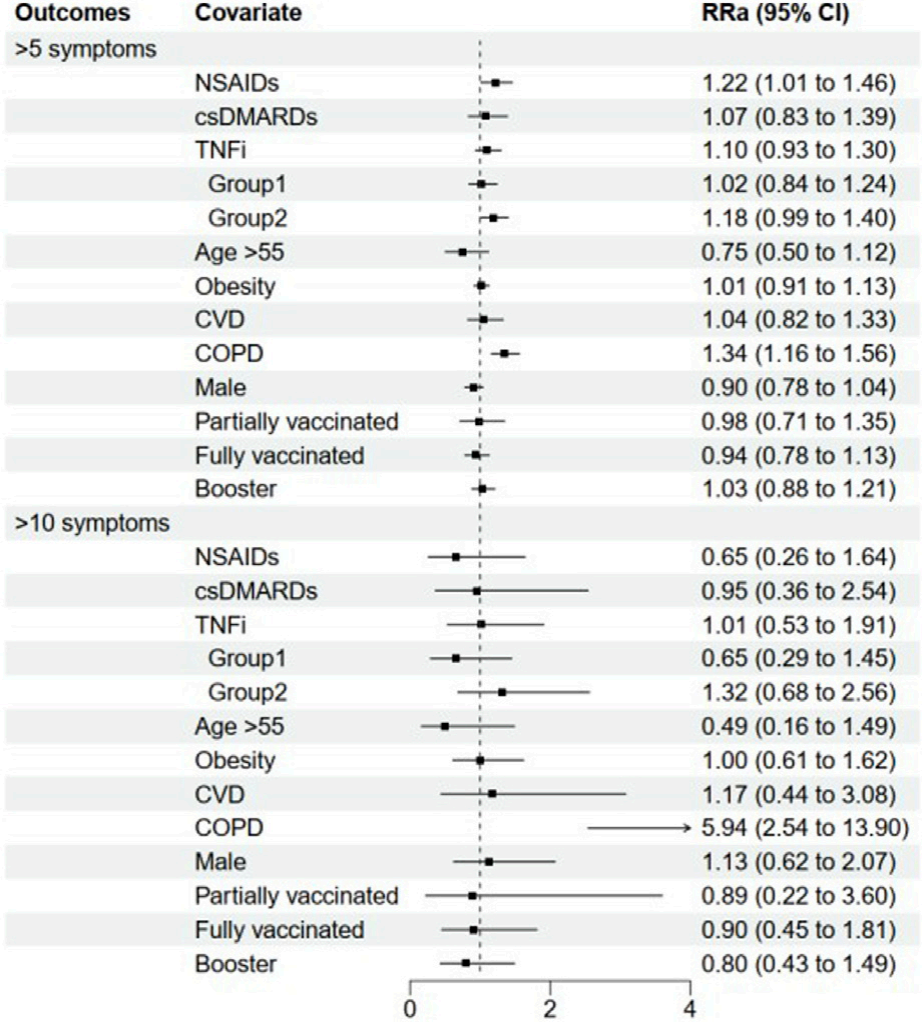
The influence of AS medications and baseline characteristics on symptom burden in mild-moderate COVID-19. Abbreviation: NSAIDs. non-steroidal anti-inflammatory drugs: csDMARDs. conventional synthetic disease-modifying antirheumatic drugs: TNFi, Tumor necrosis factor inhibitors: COPD. chronic obstructive pulmonary disease. Horizontal lines indicate the ranges of the 95% Cls and the vertical dash lines indicate the relative risk of 1. Some variables had oversized ranges of 95% CI and they were shown to be lines with arrow.

The median (IQR) number of symptoms reported by patients with no medication usage, NSAIDs monotherapy, csDMARDs monotherapy, and TNFi were 6.0 (5.0, 8.0), 8.0 (6.0, 9.0), 7.5 (5.0, 9.0), and 7.0 (5.0, 9.0), respectively (Table 2). Detailed COVID-19 symptoms are presented in Supplementary Table S1. The univariate analysis revealed that patients treated with AS medications had similar odds of experiencing more than 5 symptoms compared to those without medication use, except for patients with NSAIDs monotherapy (RR 1.26, 95% CI: 1.05–1.51). After adjusting for age, gender, obesity, smoking, alcohol drinking, coexisting comorbidities, and vaccination status, the association of csDMARDs or TNFi with the risk of experiencing more than five symptoms remained insignificant (adjusted RR (RRa) 1.07, 95% CI: 0.83–1.39 or 1.10, 0.93–1.30, respectively). However, patients with NSAIDs monotherapy still had increased odds of experiencing more than 5 symptoms (RRa 1.22, 95% CI: 1.01–1.46). Additionally, patients with COPD had a significantly increased risk of experiencing more than 5 symptoms compared to those without baseline coexisting diseases (RRa 1.34, 95% CI: 1.16–1.56) (Figure 2).

**TABLE 2:** Symptom burden and disease course of mild-moderate COVID-19 in AS with no medications, NSAIDs, csDMARDs, and TNFi.

	None (n = 85)	NSAIDs (n = 71)	csDMARDs (n = 38)	TNFi (n = 232)	TNFi subgroups	
					Group1 (n = 104)	Group2 (n = 128)
Number of symptoms	6.0 (5.0, 8.0)	8.0 (6.0, 9.0)	7.5 (5.0, 9.0)	7.0 (5.0, 9.0)	7.0 (5.0, 9.0)	8.0 (6.0, 10.0)
>5 symptoms	57 (67.1%)	60 (84.5%)	27 (71.1%)	171 (73.7%)	71 (68.3%)	100 (78.1%)
>10 symptoms	11 (12.9%)	8 (11.3%)	5 (13.2%)	31 (13.4%)	9 (8.7%)	22 (17.2%)
COVID course, days	8.0 (5.5, 13.5)	9.0 (6.0, 13.0)	9.5 (6.8, 14.5)	9.0 (6.0, 16.0)	9.0 (6.0, 14.0)	10.0 (6.0, 18.0)
LC10	35 (41.2%)	35 (49.3%)	19 (50.0%)	114 (49.1%)	45 (43.3%)	69 (53.9%)
LC28	7 (8.2%)	5 (7.0%)	3 (7.9%)	20 (8.6%)	11 (10.6%)	9 (7.0%)

Data are n (%) for categorical variables and median (IQR) for continuous variables, respectively. Abbreviation: NSAID, non-steroidal anti-inflammatory drugs; csDMARD, conventional synthetic DMARD; TNFi, tumor necrosis factor inhibitor. Group1 and 2 indicate patients with TNFi, monotherapy or combination therapy.

A similar analysis was conducted to evaluate the risk of experiencing more than 10 symptoms in AS patients. Patients treated with NSAIDs, csDMARDs and TNFi had similar odds of experiencing more than 10 symptoms compared to those without medication use in both univariable analysis and after adjusting for confounding factors (RRa 0.65, 95% CI: 0.26–1.64; 0.95, 0.36–2.54; and 1.01, 0.53–1.91 for NSAIDs, csDMARDs and TNFi, respectively). Likewise, COPD was associated with greater odds of experiencing more than 10 symptoms (RRa 5.94, 95% CI: 2.54–13.90) (Figure 2).

### 3.3 Association of AS medications with long COVID

**FIGURE 3:**
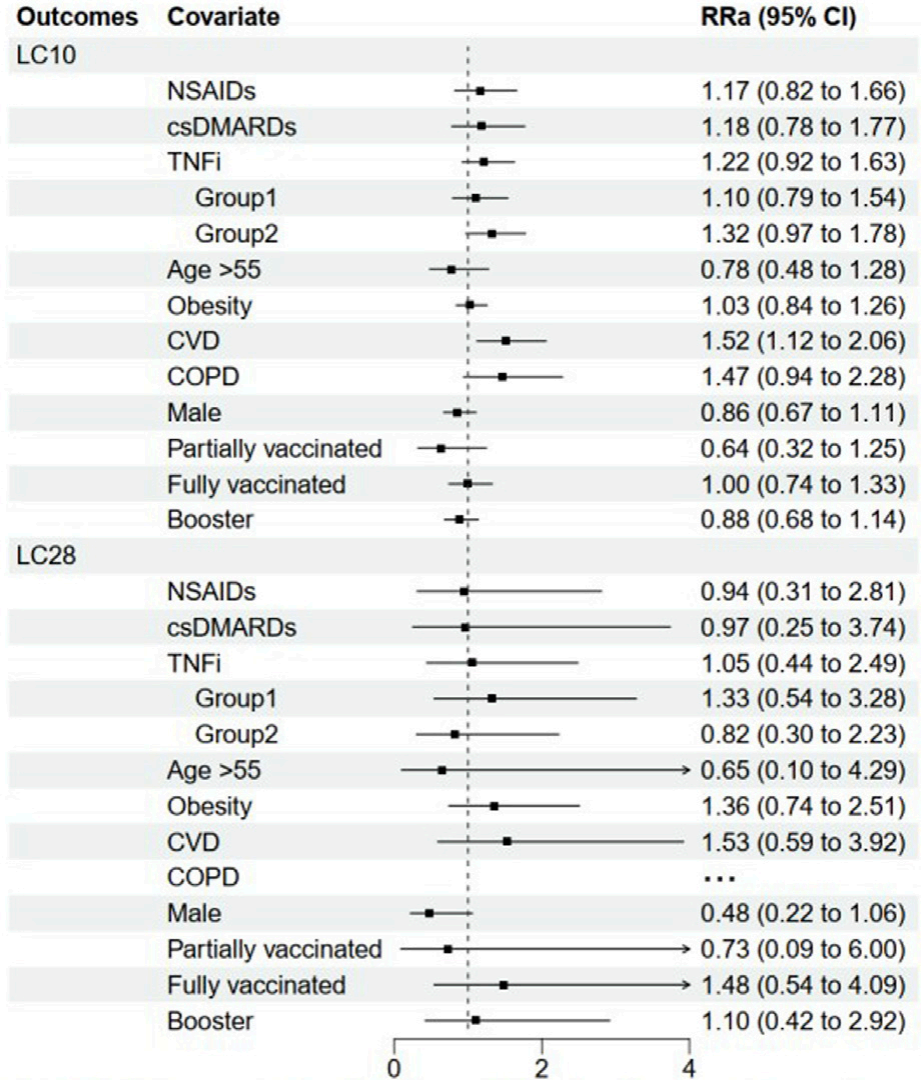
The influence of AS medications and baseline characteristics on disease course in mild-moderate COVID-19. Abbreviation: NSAIDs, non-steroidal anti-inflammatory drugs; csDMARDs, conventional synthetic disease-modifying antirheumatic drugs; TNFi, Tumor necrosis factor inhibitors; COPD, chronic obstructive pulmonary disease. Ellipsis (…) means that the model does not converge due to limited outcomes. Horizontal lines indicate the ranges of the 95% Cis and the vertical dash lines indicate the relative risk of 1. Some variables had oversized ranges 95% CI and they were shown to be lines with arrow.

The median (IQR) duration of COVID-19 symptoms in patients with no medication use, NSAIDs monotherapy, csDMARDs monotherapy, and TNFi were 8.0 (5.5, 13.5), 9.0 (6.0, 13.0), 9.5 (6.8, 14.5), and 9.0 (6.0, 16.0) days, respectively (Table 2). The univariate analysis revealed that patients with different AS medications had similar odds of long COVID (LC10) compared to those without medication use. Importantly, these associations were reproduced after adjusting for confounding factors (RRa 1.17, 95% CI: 0.82–1.66; 1.18, 0.78–1.77; and 1.22, 0.92–1.63 for NSAIDs, csDMARDs and TNFi, respectively) (Figure 3).

Similarly, when LC28 was considered as an increased disease course in COVID-19, patients with NSAIDs, csDMARDs, and TNFi had similar odds of LC28 compared to those without medication usage, both in univariate analysis and after adjustment (RRa 0.94, 95% CI: 0.31–2.81; 0.97, 0.25–3.74; and 1.05, 0.44–2.49 for NSAIDs csDMARDs and TNFi, respectively). Additionally, having cardiovascular disease (CVD) was found to be associated with greater odds of LC10 (RRa 1.52, 95% CI: 1.12–2.06), but not LC28 (RRa 1.53, 95% CI: 0.59–3.92) in the population (Figure 3).

### 3.4 Subgroup analyses by TNFi monotherapy or combination therapy

Among the 232 patients treated with TNFi, 104 patients received TNFi monotherapy (Group 1), while 128 patients were prescribed NSAIDs or csDMARDs concomitantly (Group 2). The median (IQR) number of symptoms reported by patients in Groups 1 and 2 were 7.0 (5.0, 9.0) and 8.0 (6.0, 10.0), respectively (Table 2). After adjustment for potential confounding factors, patients with TNFi monotherapy or combination therapy had similar odds of experiencing more than five symptoms compared to those without medication use (RRa 1.02, 95% CI: 0.84–1.24 and 1.18, 0.99–1.40 for Group 1 and 2, respectively). It was also revealed that combining NSAIDs or csDMARDs did not affect the odds of experiencing more than 10 symptoms in TNFi treatment (RRa 0.65, 95% CI: 0.29–1.45 and 1.32, 0.68–2.56 for Group 1 and 2, respectively) (Figure 2).

The median (IQR) duration of COVID-19 symptoms in Groups 1 and 2 were 9.0 (6.0, 14.0) and 10.0 (6.0, 18.0) days, respectively (Table 2). In multivariable analysis, patients with TNFi monotherapy or combination therapy had similar odds of long COVID (LC10) compared to those without medication use (RRa 1.10, 95% CI: 0.79–1.54 and 1.32, 0.97–1.78 for Group 1 and 2, respectively). Similar results were obtained when LC28 was used as the outcome variable (RRa 1.33, 95% CI: 0.54–3.28 and 0.82, 0.30–2.23 for Groups 1 and 2, respectively) (Figure 3).

## 4 Discussion

In this study, we examined a cohort of patients with AS and mild-moderate COVID-19. We found no association between previous use of csDMARDs or TNFi and worse COVID-19 outcomes. To our knowledge, this is the first study to investigate the impacts of csDMARDs and TNFi on AS patients with mild-moderate COVID-19. Our findings are significant as they alleviate concerns about the potential increased disease burden of COVID-19 in AS populations using csDMARDs or TNFi. These findings can potentially guide future treatment decisions.

Despite the emergence of studies reporting the characteristics, outcomes and associated factors for COVID-19 in individuals with systemic autoimmune diseases (Gianfrancesco et al., 2020; Strangfeld et al., 2021), there are limited predictive factors and prognosis analyses available specifically for AS. AS is an auto-inflammatory disease with distinct pathogenesis and treatments compared to classic autoimmune diseases. Furthermore, previous studies have focused more on severe cases of COVID-19, neglecting to fully investigate mild-moderate cases in individuals with AS, despite the fact that these cases comprised the majority of the population during the pandemic.

To assess the disease burden of mild-moderate COVID-19, we measured the number of self-reported symptoms and the duration of recovery in patients. Obviously, higher number of symptoms and longer recovery time indicate a greater burden of illness. After accounting for potential confounding factors, patients treated with csDMARDs or TNFi had similar odds of experiencing more than five symptoms compared to those not taking AS medications. The exception was patients on NSAIDs monotherapy, which presented a subtly worsening effect on symptom burden with borderline significance (RRa 1.22, 95% CI: 1.01–1.46). However, when we evaluated the outcome of having more than 10 symptoms, no significant association with COVID-19 symptoms burden was detected for all medications, both in univariable and multivariable analyses. Initially, there were concerns about the use of NSAIDs in the early stages of the pandemic due to their theoretical potential to worsen COVID-19 outcomes (Day, 2020). However, these concerns have been largely alleviated by numerous studies (Abu Esba et al., 2021; Drake et al., 2021), though the association between pre-existing NSAIDs use and COVID-19 outcomes in AS was still not fully investigated. In our study, patients on NSAIDs monotherapy had a higher percentage of COPD than patients without medication use (5.6% vs 1.2%). COPD was a known independent risk factor for severe COVID-19 (Strangfeld et al., 2021). This may affecte the association between COVID-19 symptoms and NSAIDs. Although the confounding effect of COPD was adjusted in multivariable analysis, the limited COPD cases (only 10 cases total) rendered the evaluation of its confounding effect difficult. AS disease activity may also play a role in the outcome of COVID, as patients with NSAIDs monotherapy had higher BASDAI than patients with other medications. In consideration of the possibility of confounding bias and the borderline significant effect of NSAIDs, the interpretation of this finding should be cautious and it needs re-examination in further research.

Persistent COVID-19 symptoms, also known as “long COVID,” are widespread among individuals with COVID-19 (Hopkinson et al., 2021; Davis et al., 2023). It is evident that long COVID can also occur after a mild-moderate infection, placing a greater burden on affected populations, decreasing their quality of life, and instilling fear (Davis et al., 2023). However, there is limited research on long COVID in individuals with AS, and no universally accepted definitions of long COVID in AS have been reported. In our study, the median duration of COVID-19 symptoms in the overall population was 9.0 days. Therefore, we classified patient-reported symptoms persisting for more than 10 days (LC10) as long COVID, in line with a previous study (Sudre et al., 2021). Additionally, an illness duration surpassing 28 days (LC28) was considered as another definition of long COVID. We found that the risk of LC10 was comparable for individuals using NSAIDs, csDMARDs and TNFi, compared to those not using AS medications, both in univariate analysis and after adjusting for confounding factors. Similarly, there was no significant increase in the risk of LC28 among individuals using NSAIDs, csDMARDs, and TNFi, compared to those not using AS medications. Traditionally, csDMARDs and TNFi have been associated with an increased risk of infection, although TNFi has been shown to have lower odds for severe COVID-19 outcomes (Kridin et al., 2021; Machado et al., 2023) and csDMARDs have not been found to increase the severity of COVID-19 infection in previous studies (Gianfrancesco et al., 2020; Machado et al., 2023). In line with these findings, our study demonstrates that csDMARDs and TNFi do not elevate the risk of increased symptom burden or prolonged recovery in individuals with mild-moderate COVID-19. This information may aid in the development and updating of AS management strategies during the COVID-19 pandemic.

Our study has several limitations that should be acknowledged. Firstly, there existed sampling bias due to various factors, including a single geographical area, high COVID-19 vaccination coverage (85%), a relatively young population, and limited comorbidities, which generally resulted in better COVID-19 outcomes. Secondly, in this study, patients were classified as having mild-moderate COVID-19 retrospectively. However, it should be noted that patients presenting with mild-moderate symptoms initially can progress to severe outcomes, especially among older individuals, men, and those with comorbid conditions such as cardiometabolic and pulmonary conditions (Conway et al., 2022; Kroon et al., 2022). Previous studies have examined the outcomes and characteristics of individuals with AS and severe COVID-19 (Gianfrancesco et al., 2020; Strangfeld et al., 2021; Machado et al., 2023), while these were not evaluated in our study. Therefore, caution should be exercised when interpreting our results as applicable to the entire AS population with mild-moderate COVID-19, although our sample did represent the majority. Thirdly, our study relied on self-reported laboratory results and symptoms rather than medical records to determine the occurrence and severity of COVID-19. While previous studies have reported agreement between self-reported symptoms and SARS-CoV-2 test results (Hopkinson et al., 2021), it is important to acknowledge the presence of recall bias, given the retrospective nature of this study. Moreover, we identified COVID-19 as patients with positive SARS-CoV-2 PCR or antigen test. Antigen-detecting test of SARS-CoV-2 may be less reliable than the SARS-CoV-2 PCR test, while they were both used as diagnostic methods in previous COVID-19 studies. Lastly, during the study period, the Omicron variant was the dominant SARS-CoV-2 variant in China (Sun et al., 2023). The persistence of our findings across emerging variants of SARS-CoV-2 remains unknown. However, it is worth noting that there is a general tendency for SARS-CoV-2 variants to become less virulent but more transmissible.

In conclusion, our findings suggest that the use of csDMARDs or TNFi does not result in an increased symptom burden or longer recovery time in individuals with AS following a mild-moderate COVID-19 infection. This information should be taken into account when making treatment decisions between patients and physicians.

## Data Availability

The original contributions presented in the study are included in the article/Supplementary Material, further inquiries can be directed to the corresponding authors.

## References

[B1] Abu Esba L. C. Alqahtani R. A. Thomas A. Shamas N. Alswaidan L. Mardawi G. (2021). Ibuprofen and NSAID use in COVID-19 infected patients is not associated with worse outcomes: a prospective cohort study. Infect. Dis. Ther. 10 (1), 253–268. 10.1007/s40121-020-00363-w 33135113 PMC7604230

[B2] Baimukhamedov C. (2023). How long is long COVID. Int. J. Rheum. Dis. 26 (2), 190–192. 10.1111/1756-185X.14494 36468196 PMC9878254

[B3] Calin A. Garrett S. Whitelock H. Kennedy L. G. O'Hea J. Mallorie P. (1994). A new approach to defining functional ability in ankylosing spondylitis: the development of the Bath Ankylosing Spondylitis Functional Index. J. Rheumatol. 21 (12), 2281–2285. 7699629

[B4] Carnethon M. R. De Chavez P. J. Biggs M. L. Lewis C. E. Pankow J. S. Bertoni A. G. (2012). Association of weight status with mortality in adults with incident diabetes. JAMA 308 (6), 581–590. 10.1001/jama.2012.9282 22871870 PMC3467944

[B5] Conway R. Grimshaw A. A. Konig M. F. Putman M. Duarte-García A. Tseng L. Y. (2022). SARS-cov-2 infection and COVID-19 outcomes in rheumatic diseases: a systematic literature review and meta-analysis. Arthritis. Rheumatol. 74 (5), 766–775. 10.1002/art.42030 34807517 PMC9011807

[B6] Davis H. E. McCorkell L. Vogel J. M. Topol E. J. (2023). Long COVID: major findings, mechanisms and recommendations. Nat. Rev. Microbiol. 21 (3), 133–146. 10.1038/s41579-022-00846-2 36639608 PMC9839201

[B7] Day M. (2020). COVID-19: ibuprofen should not be used for managing symptoms, say doctors and scientists. BMJ 17 (368), m1086. 10.1136/bmj.m1086 32184201

[B8] Deodhar A. Bhana S. Winthrop K. Gensler L. S. (2022). COVID-19 outcomes and vaccination in patients with spondyloarthritis. Rheumatol. Ther. 9 (4), 993–1016. 10.1007/s40744-022-00462-9 35598255 PMC9124289

[B9] Drake T. M. Fairfield C. J. Pius R. Knight S. R. Norman L. Girvan M. (2021). Non-steroidal anti-inflammatory drug use and outcomes of COVID-19 in the ISARIC clinical characterisation protocol UK cohort: a matched, prospective cohort study. Lancet Rheumatol. 3 (7), e498–e506. 10.1016/S2665-9913(21)00104-1 33997800 PMC8104907

[B10] Garrett S. Jenkinson T. Kennedy L. G. Whitelock H. Gaisford P. Calin A. (1994). A new approach to defining disease status in ankylosing spondylitis: the Bath Ankylosing Spondylitis Disease Activity Index. J. Rheumatol. 21 (12), 2286–2291. 7699630

[B11] General Office of the National Health Commission (2023). Diagnosis and treatment protocol for COVID-19 in China (trial version 10). Available at: http://www.gov.cn/zhengce/zhengceku/2023-01/06/content_5735343.htm (Accessed January 5, 2023).

[B12] Gianfrancesco M. Hyrich K. L. Al-Adely S. Carmona L. Danila M. I. Gossec L. (2020). Characteristics associated with hospitalisation for COVID-19 in people with rheumatic disease: data from the COVID-19 Global Rheumatology Alliance physician-reported registry. Ann. Rheum. Dis. 79 (7), 859–866. 10.1136/annrheumdis-2020-217871 32471903 PMC7299648

[B13] Graham F. (2023). Daily briefing: COVID-19 is no longer an international health emergency. Nature 9, 5. 10.1038/d41586-023-01577-x 37165227

[B14] Guo Y. Hu K. Li Y. Lu C. Ling K. Cai C. (2022). Targeting TNF-α for COVID-19: recent advanced and controversies. Front. Public. Health 11 (10), 833967. 10.3389/fpubh.2022.833967 PMC8873570 35223745

[B15] Hopkinson N. S. Rossi N. El-Sayed Moustafa J. Laverty A. A. Quint J. K. Freidin M. (2021). Current smoking and COVID-19 risk: results from a population symptom app in over 2.4 million people. Thorax 76 (7), 714–722. 10.1136/thoraxjnl-2020-216422 33402392

[B16] Jenkinson T. R. Mallorie P. A. Whitelock H. C. Kennedy L. G. Garrett S. L. Calin A. (1994). Defining spinal mobility in ankylosing spondylitis (AS). The Bath AS Metrology Index. J. Rheumatol. 21 (9), 1694–1698. 7799351

[B17] Kridin K. Schonmann Y. Damiani G. Peretz A. Onn E. Bitan D. T. (2021). Tumor necrosis factor inhibitors are associated with a decreased risk of COVID-19-associated hospitalization in patients with psoriasis- A population-based cohort study. Dermatol. Ther. Dermatol. Ther. 34 (4), e15003. 10.1111/dth.15003 34033207 PMC8209905

[B18] Kroon F. P. B. Najm A. Alunno A. Schoones J. W. Landewé R. B. M. Machado P. M. (2022). Risk and prognosis of SARS-cov-2 infection and vaccination against SARS-cov-2 in rheumatic and musculoskeletal diseases: a systematic literature review to inform EULAR recommendations. Ann. Rheum. Dis. 81 (3), 422–432. 10.1136/annrheumdis-2021-221575 34876462

[B19] Kun Á. Hubai A. G. Král A. Mokos J. Mikulecz B. Á. Radványi Á. (2023). Do pathogens always evolve to be less virulent? The virulence-transmission trade-off in light of the COVID-19 pandemic. Biol. Futur. 74 (1-2), 69–80. 10.1007/s42977-023-00159-2 37002448 PMC10066022

[B20] Machado P. M. Schäfer M. Mahil S. K. Liew J. Gossec L. Dand N. (2023). Characteristics associated with poor COVID-19 outcomes in people with psoriasis, psoriatic arthritis and axial spondyloarthritis: data from the COVID-19 PsoProtect and Global Rheumatology Alliance physician-reported registries. Ann. Rheum. Dis. 82 (5), 698–709. 10.1136/ard-2022-223499 36787993 PMC10176347

[B21] Nyberg T. Ferguson N. M. Nash S. G. Webster H. H. Flaxman S. Andrews N. (2022). Comparative analysis of the risks of hospitalisation and death associated with SARS-CoV-2 omicron (B.1.1.529) and delta (B.1.617.2) variants in England: a cohort study. Lancet 399 (10332), 1303–1312. 10.1016/S0140-6736(22)00462-7 35305296 PMC8926413

[B22] Strangfeld A. Schäfer M. Gianfrancesco M. A. Lawson-Tovey S. Liew J. W. Ljung L. (2021). Factors associated with COVID-19-related death in people with rheumatic diseases: results from the COVID-19 Global Rheumatology Alliance physician-reported registry. Ann. Rheum. Dis. 80 (7), 930–942. 10.1136/annrheumdis-2020-219498 33504483 PMC7843211

[B23] Sudre C. H. Murray B. Varsavsky T. Graham M. S. Penfold R. S. Bowyer R. C. (2021). Attributes and predictors of long COVID. Nat. Med. 27 (4), 626–631. 10.1038/s41591-021-01292-y 33692530 PMC7611399

[B24] Sun Y. Jin L. Dian Y. Shen M. Zeng F. Chen X. (2023). Oral Azvudine for hospitalised patients with COVID-19 and pre-existing conditions: a retrospective cohort study. EClinicalMedicine 5 (59), 101981. 10.1016/j.eclinm.2023.101981 PMC10167478 37193346

[B25] van der Linden S. Valkenburg H. A. Cats A. (1984). Evaluation of diagnostic criteria for ankylosing spondylitis. A proposal for modification of the New York criteria. Arthritis. Rheum. 27 (4), 361–368. 10.1002/art.1780270401 6231933

[B26] Wroński J. Fiedor P. (2019). The safety profile of tumor necrosis factor inhibitors in ankylosing spondylitis: are TNF inhibitors safer than we thought? J. Clin. Pharmacol. 59 (4), 445–462. 10.1002/jcph.1348 30476367

[B27] Zou G. (2004). A modified Poisson regression approach to prospective studies with binary data. Am. J. Epidemiol. 159 (7), 702–706. 10.1093/aje/kwh090 15033648

