# Supplementary material for: Do disease-modifying antirheumatic drugs and non-steroidal anti-inflammatory drugs increase the burden on ankylosing spondylitis patients with mild-moderate COVID-19? evidence from a retrospective cohort study
Source: Front Pharmacol. 2023 Oct 30;14:1266915. doi: 10.3389/fphar.2023.1266915 (PMC10642246; doi:10.3389/fphar.2023.1266915)
Supplement: Supplementary file 2 [file Table1.docx]

|  |  | AS treatment | | | | |
| --- | --- | --- | --- | --- | --- | --- |
|  | Total  (n= 521) | None  (n= 85) | NSAIDs  (n= 71) | NSAIDs & csDMARDs  (n= 95) | csDMARDs (n= 38) | TNFi  (n= 232) |
| Male sex | 420 (80.6%) | 66 (77.6%) | 55 (77.5%) | 77 (81.1%) | 26 (68.4%) | 196 (84.5%) |
| Age, years |  |  |  |  |  |  |
| 18-35 | 290 (55.7%) | 48 (56.5%) | 39 (54.9%) | 55 (57.9%) | 19 (50.0%) | 129 (55.6%) |
| 36-45 | 165 (31.7%) | 28 (32.9%) | 24 (33.8%) | 29 (30.5%) | 13 (34.2%) | 71 (30.6%) |
| 46-55 | 47 (9.0%) | 4 (4.7%) | 7 (9.9%) | 9 (9.5%) | 1 (2.6%) | 26 (11.2%) |
| ≥ 55 | 19 (3.7%) | 5 (5.9%) | 1 (1.4%) | 2 (2.1%) | 5 (13.2%) | 6 (2.6%) |
| BMI | 24.5 (22.1, 27.1) | 23.9 (21.3, 25.9) | 25.3 (22.3, 27.3) | 24.6 (22.0, 27.0) | 23.8 (21.9, 25.9) | 24.6 (22.6, 27.1) |
| Overweight | 233 (44.7%) | 31 (36.5%) | 38 (53.5%) | 42 (44.2%) | 14 (36.8%) | 108 (46.6%) |
| Comorbidities |  |  |  |  |  |  |
| None | 469 (90.0%) | 77 (90.6%) | 63 (88.7%) | 83 (87.4%) | 32 (84.2%) | 214 (92.2%) |
| Diabetes | 8 (1.5%) | 2 (2.4%) | 1 (1.4%) | 2 (2.1%) | 1 (2.6%) | 2 (0.9%) |
| CVD | 41 (7.9%) | 8 (9.4%) | 4 (5.6%) | 11 (11.6%) | 4 (10.5%) | 14 (6.0%) |
| COPD | 10 (1.9%) | 1 (1.2%) | 4 (5.6%) | 1 (1.1%) | 1 (2.6%) | 3 (1.3%) |
| Smoking status |  |  |  |  |  |  |
| None | 328 (63.0%) | 51 (60.0%) | 46 (64.8%) | 69 (72.6%) | 24 (63.2%) | 138 (59.5%) |
| Ever smokers | 193 (37.0%) | 34 (40.0%) | 25 (34.2%) | 26 (27.4%) | 14 (36.8%) | 94 (40.5%) |
| Alcohol consumption |  |  |  |  |  |  |
| None | 221 (42.4%) | 38 (44.7%) | 33 (46.5%) | 48 (50.5%) | 19 (50.0%) | 83 (35.8%) |
| With drinking habit | 300 (57.6%) | 47 (55.3%) | 38 (53.5%) | 47 (49.5%) | 19 (50.0%) | 149 (64.2%) |
| HLA-B27(+) | 442 (84.8%) | 74 (87.1%) | 59 (83.1%) | 81 (85.3%) | 30 (78.9%) | 198 (85.3%) |
| Vaccination status |  |  |  |  |  |  |
| Unvaccinated | 78 (15.0%) | 13 (15.3%) | 9 (12.7%) | 13 (12.7%) | 7 (18.4%) | 36 (15.5%) |
| Partially | 19 (3.6%) | 0 | 2 (2.8%) | 3 (3.2%) | 0 | 14 (6.0%) |
| Fully | 131 (25.1%) | 20 (23.5%) | 15 (21.1%) | 22 (23.2%) | 12 (31.6%) | 62 (26.7%) |
| Booster | 293 (56.2%) | 52 (61.2%) | 45 (63.4%) | 57 (60.0%) | 19 (50.0%) | 120 (51.7%) |
| BASDAI | 2.5 (1.2, 4.0) | 3.0 (1.6, 4.4) | 3.0 (1.4, 4.2) | 2.1 (1.0, 3.5) | 2.2 (1.2, 3.8) | 2.2 (1.2, 4.0) |
| BASFI | 1.1 (0, 3.2) | 1.2 (0.1, 4.4) | 1.8 (0.2, 4.8) | 0.6 (0, 2.7) | 1.4 (0, 2.2) | 1.1 (0, 2.9) |
| BASMI | 3.0 (0, 5.0) | 4.0 (0, 5.0) | 4.0 (0, 5.0) | 1.0 (0, 5.0) | 3.0 (0, 6.3) | 3.0 (0, 5.0) |

*Table 1:* Baseline characteristics of the participants.

Data are n (%) for categorical variables and median (IQR) for continuous variables, respectively. Percentages might not sum to 100% due to rounding.

Abbreviation: NSAID, non-steroidal anti-inflammatory drugs; csDMARD, conventional synthetic DMARD; TNFi, tumor necrosis factor inhibitor;

BMI, body mass index; COPD, chronic obstructive pulmonary disease; cardiovascular disease (CVD, including hypertension).

|  | None  (n= 85) | NSAIDs  (n= 71) | csDMARDs  (n= 38) | TNFi  (n= 232) | TNFi subgroups | |
| --- | --- | --- | --- | --- | --- | --- |
|  |  |  |  |  | Group1 (n= 104) | Group2 (n= 128) |
| Number of symptoms | 6.0 (5.0, 8.0) | 8.0 (6.0, 9.0) | 7.5 (5.0, 9.0) | 7.0 (5.0, 9.0) | 7.0 (5.0, 9.0) | 8.0 (6.0, 10.0) |
| > 5 symptoms | 57 (67.1%) | 60 (84.5%) | 27 (71.1%) | 171 (73.7%) | 71 (68.3%) | 100 (78.1%) |
| > 10 symptoms | 11 (12.9%) | 8 (11.3%) | 5 (13.2%) | 31 (13.4%) | 9 (8.7%) | 22 (17.2%) |
| COVID course, days | 8.0 (5.5, 13.5) | 9.0 (6.0, 13.0) | 9.5 (6.8, 14.5) | 9.0 (6.0, 16.0) | 9.0 (6.0, 14.0) | 10.0 (6.0, 18.0) |
| LC10 | 35 (41.2%) | 35 (49.3%) | 19 (50.0%) | 114 (49.1%) | 45 (43.3%) | 69 (53.9%) |
| LC28 | 7 (8.2%) | 5 (7.0%) | 3 (7.9%) | 20 (8.6%) | 11 (10.6%) | 9 (7.0%) |

*Table 2:* Symptom burden and disease course of mild-moderate COVID-19 in AS with no medications, NSAIDs, csDMARDs, and TNFi.

Data are n (%) for categorical variables and median (IQR) for continuous variables, respectively.

Abbreviation: NSAID, non-steroidal anti-inflammatory drugs; csDMARD, conventional synthetic DMARD; TNFi, tumor necrosis factor inhibitor. Group1 and 2 indicate patients with TNFi monotherapy or combination therapy.
